# COVID-19 as an Immune Complex Hypersensitivity in Antigen Excess Conditions: Theoretical Pathogenetic Process and Suggestions for Potential Therapeutic Interventions

**DOI:** 10.3389/fimmu.2020.566000

**Published:** 2020-10-21

**Authors:** Giovanni Manzo

**Affiliations:** Chemical-Biological Department, Istituto di Istruzione Secondaria Superiore (IISS) ‘E. Lanoce’, Maglie, Italy

**Keywords:** Covid-19, systemic disorders, spike antigen, ACE-2 receptor, anti-spike antibodies, complement activation, Kawasaki-like disease

## Abstract

Because of particular properties of SARS-Cov-2, such as an high infection speed, its antigenic nature, evolutionarily unknown to the human immune system, and/or a viral interference on the immune response mechanisms, this virus would determine in the subjects a delayed anomalous (slow and/or low) immune response, ineffective and, finally, self-damaging. The hypothetical pathogenetic process for covid-19 could occur in three phases: a) Viral phase, asymptomatic or weakly symptomatic, with an a-specific innate immune response; b) Immunological phase, intermediately symptomatic, with an anomalous specific immune response (delayed, slow and/or low synthesis of IgM and IgG) in antigen excess conditions, immune complex formation and complement activation with tissue damages; c) Hemo-vascular phase, severely symptomatic, where complement-mediated tissue damages would induce vascular inflammation and systemic alteration of the coagulation homeostasis. This hypothesis is well supported by the immune-histochemical and microscopic demonstration in severe patient lungs of co-localized spike viral proteins, terminal components of the activated complement system (C5b-9 membrane attack complex) and microvascular deposits of small fibrin thrombi. This picture could be aggravated by the involvement of neutrophils and macrophages, releasing additional lytic and inflammatory factors. Thus, covid-19 would arise as a simple viral infection, develop as a diffuse immune complex hypersensitivity and explode as a systemic hemo-vascular pathology. If this hypothesized process would be real, suitable therapeutic interventions might be carried out, able to interfere with or block the critical factors in the various phases.

## Introduction

Recent data about therapeutic effects of “tocilizumab” (an anti-IL-6 mAb utilized in rheumatoid arthritis) in covid-19 cases, induce to hypothesize that the SARS-cov-2 pathology is due to endogen biological mechanisms, rather than to specific viral activities. Thus, it could be hypothesized that the pathogenetic mechanism in arthritis and covid-19 might have commons or correlated points. Likely, IL-6 might be a such point. From data well established in the scientific literature, it is known that IL-6 is an important factor of the immune response, released from the beginning by macrophages, the activity of which then involves neutrophils, B lymphocytes and antibody (Ab) synthesis, as well as, complement (C) activation in the classical way, with release of inflammatory factors (anaphylatoxins) and consequent potential lesions of the tissues where the target antigens (Ag) are located.

## Hypothesis of COVID-19 as Immune Complex Hypersensitivity in Antigen Excess Conditions

It is known that in diseases such as rheumatoid arthritis, the pathological effect is the result of immune reactions occurring “*in vivo*” in an excess of Abs, that determine formation of soluble Ag-Ab immune complexes (IC). These, being no removable by phagocytes, remain in circulation, settle and persist in defined tissue places (such as articulations), where they induce reiterative C activation in the classical way, with persistent inflammation and increasing tissue damages. I speculate that something similar might occur in covid-19, for a synergic convergence of various factors with a viral, biological and environmental nature. Main viral factors would be a high infecting load, a very fast growth-propagation and an antigenic nature evolutionarily unknown to the human immune system (IS), as well as a possible viral interference on the immune response mechanisms. These factors would lead the IS to realize an anomalous immune response, delayed, slow and/or weak, favoring an increasing viral growth determining Ag excess conditions. This would cause IC formation and, thus, immune reactions involving a repeated C activation in the classical way, with persistent inflammation and tissue lesions, in a typical picture of IC hypersensitivity. This pathological picture could be strongly aggravated, made persistent and potentially lethal, by environmental pollutants. Stable virus adsorption on pollutant particles might lead to an increasing local virus concentration, able to induce a sort of “persistent viremia”, that, long time, is known to determine a typical picture of IC hypersensitivity, where the classically activated complement cascade would be the crucial disease factor. Thus, it might be hypothesized that, in the absence of a very high viral load, the covid-19 course might normally evolve in a more or less severe pathological picture, solvable by natural and/or pharmacological factors. But, on the contrary, it can explode when other biological or environmental factors concentrate the virus, favoring Ag excess conditions and, thus, massively triggering the aforesaid hypersensitivity mechanisms.

## Theoretical Pathogenetic Process for COVID-19

Several recent emerging reports, strongly supporting the hypothesis that complement activation is related with the pathogenesis of covid-19, lead to suggest the following global scenario for this disease, that would develop in three distinct phases: viral, immunological and hemo-vascular.

- Viral phase: early, the virus infection would localize in the high respiratory tracts, causing initial weak symptoms and inducing a non-specific innate immune response, through germline pattern recognition receptors (PRRs) detecting pathogen-associated molecular patterns (PAMPs). This response immediately involve phagocyte cells (macrophages, monocytes, dendritic cells, neutrophils), release of cytokines (IL-1, IL-6,TNFa), activation of the complement (C) system by alternative and lectin pathways (1, 2), C3d factor production and consequent B lymphocyte and neutrophil attraction “in loco.” The result of this phase might be the virus elimination if the infecting load is low and the subject innate immune response is effective, or the virus persistence and proliferation, if the viral load is high and the subject innate immune response is ineffective.
- Immunological phase: the virus replication and propagation to the middle/low respiratory ducts *via* ACE-2 receptors (ACE-2R) (3) would cause intermediate symptoms and induce the specific immune response, in particular a humoral response, with involvement of B lymphocytes. Because of the peculiar immunological nature of the viral spike (S) antigen, as an evolutionarily unknown target for the human immune system (IS), and the high infection-growth speed of the virus, the immune response would be anomalous. Namely, Ab production could result absent, delayed or slow for IgM synthesis and anticipated or low for IgG synthesis (4, 5). In such a way, the virus would fast replicate, determining an Ag excess condition “*in vivo*” and a consequent formation of soluble Ag-Ab-Ag ICs. They could remain in circulation and settle on various tissue sites, particularly in the capillary endothelium (2, 6) of several organs, including lungs, heart, kidneys, brain, skin. Here, ICs would bind the C1q factor, thus triggering the classical C system pathway. In this process, release of C4a, C3a and C5a anaphylatoxins would determine mast cell degranulation with histamine release, generating a “cytokine storm,” thus promoting a systemic pro-inflammatory immune response. Consequent attraction and activity of neutrophils and monocytes/macrophages, together with the C5b-9 membrane attack complex (MAC) activity, would cause cell lysis and severe tissue damages (1, 2, 7, 8). Since pulmonary alveoli are rich in ACE-2Rs, as targets of the viral S glycoproteins, the lungs would be the early damaged organs, but then a systemic involvement of different organs would occur at the level of their capillaries, also very rich in ACE-2Rs. The final result of this phase could be a symptom regression if a timely, quantitative and qualitative suitable Ab production leads, in some a way, to a low/absent IC formation, otherwise a symptom progression if a high IC formation occurs. In addition to soluble circulating ICs, other ICs directly fixed on the cell surfaces could form at the sites of ACE-2Rs. In fact, since ACE-2Rs and anti-S Abs are competitors for the RBD (receptor binding domain) present at the top of the S1 subunit of spikes, and since some anti-S Abs are able to bridge two separate RBDs (9), ICs of the ACE-2R/RBD/Ab/RBD/ACE-2R type could directly form on the cell surfaces, immediately activating the C1q and the C classical pathway until the C5b-9 MAC, with consequent tissue damages.
- Hemo-vascular phase: this phase would be a direct immediate consequence of the C-mediated tissue damages occurred in the immunological phase. It is well known that, in normal conditions, the blood coagulation homeostasis is the result of a multifactorial equilibrium between the fibrin-synthesis and fibrin-lysis processes, thanks to a physiological release in circulation of tissue-platelet factors (TF, PF) and tissue plasminogen activator (TPA), respectively regulating the two processes, together with several other factors. Now, when a tissue damage occurs, TFs and PFs immediately are released in circulation and the aforesaid balance naturally shifts toward the fibrin-synthesis process for controlling an eventual hemorrhagic status. Thus, C-mediated damages of the capillary endothelium would directly lead to a microvascular pro-coagulant thrombogenic status (1, 8), that, from the emerging experimental reports, seems to be the main systemic pathological feature of the covid-19 disease. This scenario could be aggravated by pollutants, whose presence “in loco” and entrance the circulation could induce systemic neutrophil and macrophage activity, with a consequent release of their inflammatory and lytic factors, further worsening the clinical picture in severe covid-19 patients (1, 2, 10–12).

## Discussion

- The hypothesized IC formation in Ag excess conditions is an event that, in nature, does not occur for antigenic structures evolutionarily known to the IS. Nevertheless, in some “persistent viremia” (and covid-19 might become such in the organism, owing to the aforesaid factors), ICs can continuously form, causing chronic inflammatory lesions in blood capillaries. This is what occurs in defined artificial immune reactions, such as the “serum disease,” where a given substance, inoculated in large amount, act early as an immunogen and then as a reactive antigen, just in Ag excess conditions. It seems of interest and important the fact that, in the “serum disease,” perivascular inflammatory lesions occur in the renal glomeruli and coronaries after 7 to 14 days, with concomitant phenomena of permeability increase (extravasation), thrombus formation, C activation and massive PMN infiltration: an histopathological picture very similar to that of the covid-19 syndrome (1, 10).
- With regard to the aspects of “immunization” arising in the covid-19 process, recent laboratory data (4) indicate that: 1) In a study on the Ab responses to SARS-Cov-2, all the covid-19 patients tested (285 = 100%) resulted positive for antiviral IgG Abs within 19 days after the symptom onset, while the proportion of patients with antiviral IgM Abs only reach about 94% after 20 to 22 days (4). 2) The levels of IgG and IgM in several patients reach their peak about 8 to 14 days after the symptom onset, with the IgG level simultaneous or slightly earlier and higher than IgM level. This appears to be anomalous for a canonical immune response, where usually IgM synthesis is earlier and higher than that of IgG (4, 5). 3) In 26 patients initially seronegative, during the observation period, three seroconversion types occurred: synchronous IgM/IgG (9 patients), earlier IgM (7 patients) and earlier IgG (10 patients): in all the cases, IgG level is always higher than IgM level, as previously observed in immune responses to SARS-coronavirus (5), where it is unclear if some patients have an anomalous primary immune response (IgM delayed or absent synthesis) or an anticipated secondary immune response (IgG accelerated synthesis) (4, 5).
- IgM and IgG titers in a severe patient group were higher (mainly for IgG) than those in a non-severe patient group, 2 to 3 weeks after the symptom onset. Previous observations suggested that an IgG upsurge for SARS-CoV correlates with a clinical worsening of pneumonia (5). In a pre-print study, SARS-Cov2 neutralizing Ab responses result more robust in 35 patients with severe disease, about 1 month after infection (13). Moreover, in five patients with severe Covid-19, three potent neutralizing antibodies against multiple epitopes on the SARS-Cov2 spike have been detected: one anti-RBD, one other anti-NTD (N-terminal domain) and a third able to bind two separate RBD (9). These data, all occurring in severe disease patients, could suggest that, before the Abs become protective against the virus, they could constitute an initial crucial factor of disease. More in particular, the aforesaid third Ab type might bridge separate RBDs on spikes already bound to ACE-2Rs on the cell surfaces, generating ACE-2R/RBD/Ab/RBD/ACE-2R complexes, equivalent to ICs already fixed on cell surfaces, and thus able to directly activate C1q and the C classical pathway until C5b-9 MAC. This events might advance until the Ab titer becomes high enough to prevent spikes from binding ACE-2Rs. To this regard, the question could arise about the effect of anti-hypertensive drugs involving ACE-2 (ACE-2 inhibitors) or ACE-2R (sartans): theoretically, blocking the conversion AT1/AT2 by ACE-2, the ACE-2 inhibitors would increase the amount of ACE-2Rs free for spike, thus favoring the disease; on the contrary, sartans, competing with spike for ACE-2Rs would decrease the number of free receptors for spike, thus hindering the disease. This might be supported by data of a report of the Italian ISS, where among 1102 covid-19 individuals with pre-existent pathologies, 27% of dead patients used ACE inhibitors, and 16% sartans as therapy (14).
- Many of the aforesaid highly neutralizing Abs, unexpectedly, show V(D)J sequences close to germline sequences, without extensive somatic hyper-mutations (9). Since it is known that the somatic hyper-mutations generate many different B-cell clones, whose Abs bind the Ags with a highly variable affinity, could these hyper-mutated Abs have Fabs unable to react adequately with the viral Ags, so that their Fc piece assume a configuration not suitable to bind C1q? Otherwise, could they be unable to form circulating or fixed ICs? If so, could these be hypothetical reasons for which only some of the patients, just those producing potent germline neutralizing anti-RBD Abs, would become severe patients, which might, then, heal or die depending on the timely, qualitative and quantitative Ab synthesis?
- Well, in Covid-19, the immune response appears to be often anomalous. Could this be due to genetic or epigenetic viral interferences on the isotype IgM/IgG switch and lymphocyte affinity maturation? On this matter, it is known that some alterations of the lymphocyte maturation (such as hyper IgM syndrome, a-gamma-globulinemia and some SCID forms) are linked to genes on the X chromosome, where ACE-2, immunity, inflammation, and coagulation genes are also located (15). Thus, the doubt could arise that the anomalous immune response in covid-19 might also be due to a viral interference on such human genes. If so, an explanation might be given to the fact that, in covid-19, severe patients are about 2/3 males and 1/3 females (14): presumably, the presence of 2 X-chromosomes in females might protect the immune response functions better than 1 X-chromosome in males, thanks to the mosaic activity of both maternal and paternal X chromosomes (15).
- Therapeutic use of serum Abs from healed subjects (plasma therapy) might have positive effects: recent unofficial data would indicate that a therapy with hyper-immune sera in the initial covid-19 appears to prevent the disease progression, while for patients in advanced severe disease a significant improvement has been noted. This could indicate that Abs might have a favorable effect, but, presumably, only if in high quantities (13), as it occurs in hyper-immune sera therapy. In effect, besides a direct neutralization of viral activities, such as replication, that reduces the viral titer, high Ab amounts might also act leading the Ag/Ab rate closer to the equivalence, that reduce or avoid formation of soluble and fixed ICs. On the contrary, insufficient quantities of Abs (as often it occurs early in the covid-19 patients) (4) might sort an unfavorable increase of ICs and C activity. Thus, in covid-19, the use of mFabs or artificial micro-Abs could be more suitable than the use of whole Abs. In fact, it is shown that they can neutralize viral Ags (16) without C activation and without the undesired effects of heterologous sera in plasma therapy.
- Thus, I think that, in covid-19, the C activation by ICs might be the crucial point of disease. This hypothesis might be supported by recent observations that only a low occurrence of allergic asthmatics is present among admitted covid-19 cases (3 out of 275 individuals) (17). If so, this would be just in agreement with the proposed hypothesis, since at the secondary immune response in allergic subjects, IgG synthesis does not occur, whereas synthesis occurs of IgE, which are unable to bind C on their Fc piece. It is known that human IgG1-2-3 are highly reactive for the C, while IgG4 are not reactive: thus, it would be of interest to know if some correlations in the various patients could exist among their IgG class, C activation and a different covid-19 evolution. Moreover, it could be interesting to see if the anti-spike Abs endowed with hyper-mutated V(D)J sequences have a molecular structure unable to bind Ags in a correct way conferring to the Fc piece the configuration suitable for activate C1. If so, could an allergic condition (IgE synthesis) and hyper-mutated Abs be protective, while Abs with germline sequences favor the disease?
- The idea that C activation would be the crucial point in covid-19 is strongly supported by several reports about the C role in the Covid-19 disease (1, 2, 7, 10). In particular, it has been shown that: 1) The C activation contribute to the covid-19 pathogenesis regulating a systemic pro-inflammatory response: in a mouse model, mice deficient in C3 (C3^−/−^), the central component of the C system, exhibited significantly less respiratory disorders, fewer neutrophils and inflammatory monocytes, lover cytokine levels to C56BL/6J control mice, despite similar viral titers detected in the lungs (2). 2) Although the alternative and lectin C pathways are also involved, the classical C pathway activation would appear mainly responsible for the Covid-19 pathogenesis, as the release of 4a, 3a, 5a anaphylatoxins would indicate (1). Moreover, deposition of C components of the classical pathway has been observed in the lungs of SARS-Cov infected mice (2). 3) The C5b-9 MAC and C4d factor have been detected as deposits in skin lesions and alveolar micro-vasculature, as well as in normally appearing skin of patients (1). Again, co-localization of SARS-Cov-2 spike glyco-proteins with C4d and C5b-9 MAC in inter-alveolar septal capillaries and cutaneous microvasculature of some covid-19 patients examined has been shown (1). 4) Such a co-localization of viral spike Ags with C components necessarily implies that Ag-Ab ICs are settled or directly formed in those structures, strongly supporting the hypothesis that covid-19 would be an immune complex hypersensitivity in antigen excess conditions, and that the C cascade induced by the ICs is responsible for severe tissue lesions (1).
- In concomitance with C activation, the following pathological features have been detected in the different organs of severe patients: a systemic pro-coagulant state with generalized micro-vascular thrombosis, fibrin deposition in inter-alveolar septa and alveolar spaces, fibrin and thrombi in capillaries, red cell extravasation, neutrophil and monocyte collection in the septa and alveolar spaces, extensive endothelial and sub-endothelial deposits of C5b-9 complexes in thrombosed arteries (1). Because of these pathological features in severe Covid-19 patients, therapeutic interventions have been proposed utilizing anti-complement components (C3a, C5a) and/or anti-coagulant drugs (1, 8, 10).
- Recently, a pre-print paper (18) has reported number reduction and function exhaustion of T-cells in Covid-19 patients, directly correlated with the age and the clinical severity of the subjects, and inversely correlated to the serum concentration of IL-6, IL-10, and TNF-a cytokines (18). Significantly, these T-cells (CD8+ and CD4+) show an increasing expression of PD-1 and TIM3 markers during the worsening of the disease. Since TIM3 and PD-1 are related to cell exhaustion and apoptosis, one could speculate that a T-cell response might be useful in the initial viral phase of the disease, for destroying infected host cells and blocking the virus replication. But, then, when a B-cell humoral response becomes dominant, T-cells would be unnecessary or ineffective, and thus would become exhausted and/or apoptotic.
- The Kawasaki disease (KD) is a vasculitis of the children associated to concurrent respiratory viruses (19, 20). Recent laboratory data suggest a causal relationship between Covid-19 and a Kawasaki-like hyper-inflammatory syndrome (21), presumably as a consequence of a post-viral immunological reaction, that could result in an acute myocarditis associated to an high IgG serum rate (22). This picture seems to be similar to that of a “persistent viremia,” where a condition of “pseudo-tolerance” of the virus by the host induces continuous hyper-production of IgG, thus causing an Ab excess that lead to formation of ICs responsible for the vasculitis and organ damages. This mechanism could be similar to that of some post-streptococcus diseases, where organ damages occur when the pathogen agent is already absent or present only in small foci. In conclusion, some important question could arise: a) Could the K-like disease in the children to be a sort of IC hypersensitivity due to a persistent viremia? b) Could Covid-19 in adults become a persistent post-disease infection inducing a continuous Ab production and IC formation responsible for tardive post-covid-19 pathologies, as in the children K-like disease? To this end, a screening of Covid-19 ex-patients could be opportune for detecting an eventual serum presence of ICs. 3) If so, could Covid-19 arise as a hypersensitivity disease in Ag excess conditions and become, then, a hypersensitivity disease in Ab excess conditions?

## Conclusions and Suggestions for Potential Therapeutic Interventions

In conclusion, the covid-19 syndrome would arise as a respiratory persistent viral infection, develop as a diffuse IC hypersensitivity and finally explode as a systemic micro-vascular injury. Therefore, if the pathogenetic process above proposed for Covid-19 is real, the following therapeutic interventions, targeting to block or control the critical factors in the progressive consequent phases, might be tested, as schematically suggested in **Table 1**. Note that the therapeutic interventions for the second phase might be opportunely associated to those of the first phase, as well as interventions for third phase might be associated to those of the precedent phases, in giving manner, doses and times to be defined.

**Table 1:** Potential Anti-Covid-19 Therapeutic Treatments.

PHASES AND SYMPTOMS	THERAPEUTIC TARGETS	THERAPEUTIC INTERVENTIONS
**1) VIRAL PHASE ** (about 1–5° days) Inflammation of first air ducts and eyes (rhinitis, pharyngitis, conjunctivitis). Inflammation of middle air tracts (tracheitis, bronchitis, fever, cough)	- Blocking inflammation factors and histamine (amplifying inflammatory effects) - Neutralizing the virus for preventing its replication and a consequent Ag excess condition	- Anti-inflammation drugs - Anti-histamine drugs - Anti-Spike (RBD, NTD) mFabs
**2) IMMUNOLOGICAL PHASE** (about 6–10° days) Inflammation of the lower air tracts and pulmonary alveoli, dyspnea, tissue and vascular C-mediated lesions	- Blocking the IC formation by neutralization of the viral Ags without involving the C system - Blocking the complement cascade activation and its systemic effects	-Anti-spike(RBD) mFabs or -Anti-spike(RBD) micro-Abs - C1 INH (inhibitor of C1), already in use as a drug for hereditary angioedema and other extravasation diseases
**3) HEMO-VASCULAR PHASE** (about 11–15° days and more) Tissue and vascular lesions, thrombus formation, extravasation (interstitial pneumonia, renal, heart, brain, and skin damages)	- Preventing platelet aggregation and activation - Blocking the fibrin synthesis process -Dissolving the systemic micro- vascular thrombi	- Acetyl salicylic acid - Anti-histamine drugs - Anti-coagulant drugs - Fibrin-lytic agents

## Data Availability Statement

The original contributions presented in the study are included in the article/supplementary material; further inquiries can be directed to the corresponding author.

## Author Contributions

The author confirms being the sole contributor of this work and has approved it for publication.

## Conflict of Interest

The author declares that the research was conducted in the absence of any commercial or financial relationships that could be construed as a potential conflict of interest.
